# A Modified Model Dielectric Function for Analyzing Optical Spectra of InGaN Nanofilms on Sapphire Substrates

**DOI:** 10.3390/nano15070485

**Published:** 2025-03-24

**Authors:** Devki N. Talwar, Hao-Hsiung Lin, Jason T. Haraldsen

**Affiliations:** 1 Department of Physics, University of North Florida, 1 UNF Drive, Jacksonville, FL 32224-7699, USA; j.t.haraldsen@unf.edu; 2 Department of Physics, Indiana University of Pennsylvania, 975 Oakland Avenue, 56 Weyandt Hall, Indiana, PA 15705-1087, USA; 3 Graduate Institute of Electronics Engineering and Department of Electrical Engineering, National Taiwan University, Taipei 10617, Taiwan; hhlin@ntu.tw.edu

**Keywords:** optical constants of InN, GaN, reflectivity, absorption and transmission spectra, InxGa1−xN/Sapphire film thickness, model dielectric function, transfer matrix method

## Abstract

Due to a lower InN bandgap $energy\;E_{g}\sim 0.7\;eV$, ${In}_{x}{Ga}_{1-x}N/Sapphire$ epifilms are considered valuable in the development of low-dimensional heterostructure-based photonic devices. Adjusting the composition x and thickness d in epitaxially grown films has offered many possibilities of light emission across a wide spectral range, from ultraviolet through visible into near-infrared regions. Optical properties have played important roles in making semiconductor materials useful in electro-optic applications. Despite the efforts to grow ${In}_{x}{Ga}_{1-x}N$/Sapphire samples, no x- and d-dependent optical studies exist for ultrathin films. Many researchers have used computationally intensive methods to study the electronic band structures $E_{j}\left(\vec{k}\right),$ and subsequently derive optical properties. By including inter-band transitions at critical points from $E_{j}\left(\vec{k}\right)$, we have developed a semiempirical approach to comprehend the optical characteristics of InN, GaN and ${In}_{x}{Ga}_{1-x}N$. Refractive indices of ${In}_{x}{Ga}_{1-x}N$ and sapphire substrate are meticulously integrated into a transfer matrix method to simulate d- and x-dependent reflectivity $R\left(E\right)\;$ and transmission $T\left(E\right)$ spectra of nanostructured ${In}_{x}{Ga}_{1-x}N/Sapphire$ epifilms. Analyses of $R\left(E\right)$ and $T\left(E\right)$ have offered accurate x-dependent shifts of energy gaps for ${In}_{x}{Ga}_{1-x}N$ (x = 0.5, 0.7) in excellent agreement with the experimental data.

## 1. Introduction

Binary III-nitrides (XN) and their ternary $X_{x}Y_{1-x}N$, and quaternary $X_{x}Y_{1-x-y}Z_{y}N$ alloys (with X,Y,Z≡Al,Ga,In) have emerged as some of the most prominent universal materials [1,2,3,4,5,6,7,8]. As compared to the conventional group III–V semiconductors (e.g., ${In}_{x}{Ga}_{1-x}As$, ${In}_{x}{Ga}_{1-x}P$, etc.) [8], the III-Ns are less toxic and more abundant in nature. Before 2003, the widely accepted value of the electronic energy bandgap ($E_{g}$) for the hexagonal or wurtzite (wz) InN was projected in the range of 1.9 eV to 2.3 eV [9,10,11]. Based on recent absorption and photoluminescence (PL) measurements, many experimentalists [12,13,14] have challenged these large energy bandgaps and validated a much smaller $E_{g}$ from 0.67 eV to 0.75 eV, at room temperature (RT). Inconsistencies in the higher values of $E_{g}$ were attributed primarily to the difficulties of growing better-quality wz InN films [9,10,11,12,13,14]. Moreover, the larger bandgaps established earlier for InN samples were prepared using sputtering techniques compared to those grown by molecular beam epitaxy (MBE) [12,13,14]. Today, the smaller bandgap of wz InN (around ~0.7 eV) has been broadly accepted to be explicitly reliable. Density functional theory (DFT) [15,16,17,18] and full-potential linearized augmented plane-wave (FP-LAPW) methods implemented in the WIEN2K software have also substantiated the smaller $E_{g}$~0.7 eV for binary wz InN material [16,17,18,19]. Ever since the revision made in the lower energy bandgap, different InN-based hexagonal ternary (e.g., ${In}_{x}{Ga}_{1-x}N,and{In}_{x}{Al}_{1-x}N)$ alloys have gained tremendous attention from the academic and engineering community [20,21,22,23,24,25,26,27,28,29,30,31,32,33,34,35,36,37,38,39,40,41,42,43,44,45,46,47,48,49].

**Figure 1:**
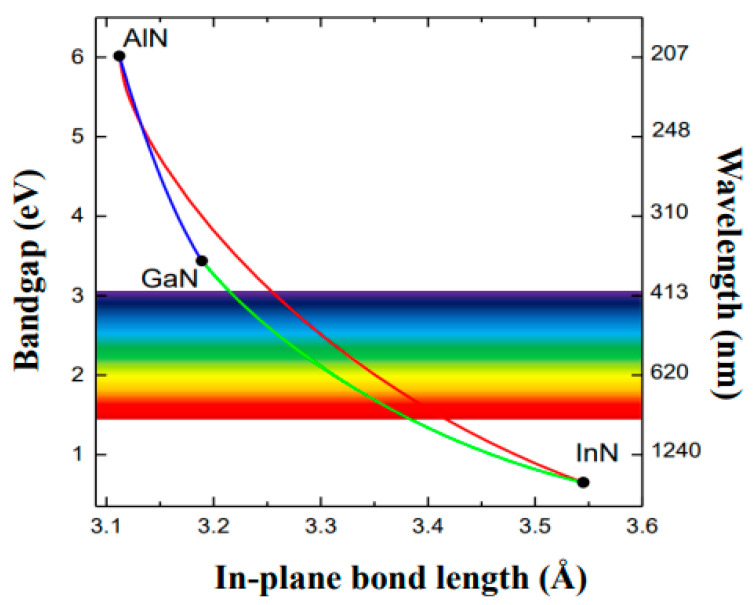
The relationship of in-plane bond length (Å) is displayed with their energy bandgaps $E_{g}$ (eV) and photonic wavelengths λ (nm) for the conventional wurtzite III-N materials.

Among other III-N (${Al}_{x}{Ga}_{1-x}N,{Al}_{x}{In}_{1-x}N$) alloys, ${In}_{x}{Ga}_{1-x}N$ is considered extremely valuable for designing various optoelectronic devices [1,2,3,4,5,6,7,8]. A major advantage (see Figure 1) of ${In}_{x}{Ga}_{1-x}N$ alloy has been the ability to adjust direct bandgaps $E_{g}$ between InN (~0.7 eV) and GaN (3.42 eV) materials by varying the In composition, x [20,21,22,23,24,25,26,27,28,29,30,31,32,33,34,35,36,37,38,39,40,41,42,43,44,45,46,47,48,49].

This tuning of x in ${In}_{x}{Ga}_{1-x}N$ has allowed regulating the emission of light across the visible spectrum, from blue to green and even into the yellow range [20,21,22,23,24,25,26,27,28,29,30,31,32,33,34,35,36,37,38,39,40,41,42,43,44,45,46,47,48,49]. The alloy is also characterized by high thermal stability, saturated velocity, high electron mobility, large spontaneous electric field, with excellent radiation resistance, polarization-induced high electron sheet charges [40,41,42,43,44], etc.

These characteristics have motivated many scientists and engineers to design infrared photodetectors, high electron mobility transistors (HEMTs), light-emitting diodes (LEDs), laser diodes (LDs), ultraviolet (UV) lasers, near-infrared (NIR)/UV photodetectors, night vision systems, thermal imaging cameras, mid-infrared lasers, high-efficiency multi-junction solar cells [16,17,18,19], etc. Many such devices are being integrated into high-performance optoelectronics to achieve the next generation of IR-based modules for optical communication (λ = 1.55 μm) networks [18,19]. Nanoelectronics has also been considered for attaining thermal management systems to monitor high power, high voltage and high temperature [18,19] in different commercial production sites, including power generation, aerospace, nuclear, electric automobiles, oil/gas exploration, etc.

Despite many positive attributes, designing different device structures based on good-quality epifilms has been a problem. Achieving In-rich ${In}_{x}{Ga}_{1-x}N$ alloys and the formation of phase separations have caused additional challenges [18] for crystal growers. These issues are linked to the material’s low dissociation temperature and the lack of suitable substrates [18,19]. The growing needs of low-dimensional heterostructures (LDHs) (i.e., multi-quantum wells (MQWs) and superlattices (SLs)) requiring ultrathin ${In}_{x}{Ga}_{1-x}N$ films with appropriate composition, x, and thickness, d, have motivated many scientists to prepare them on sapphire (Al_2_O_3_) substrate. Suitable growth techniques are adopted depending on the required features for device designs. The methods that are commonly used to obtain ${In}_{x}{Ga}_{1-x}N$ films include metal–organic chemical vapor deposition (MOCVD) [45,46,47], MBE, plasma-assisted (PA) MBE [48,49,50,51,52,53,54,55,56,57,58], remote-plasma-enhanced or reactive-rf sputtering chemical vapor deposition (RPCVD), high-pressure CVD (HPCVD), hydride vapor phase epitaxy (HVPE), [45,46,47,48,49,50,51,52,53,54,55,56,57,58] etc. To achieve good-quality ${In}_{x}{Ga}_{1-x}N$ epifilms, the required elements (In, Ga and N) are supplied in gaseous or atomic forms in appropriate growth chambers onto the sapphire surfaces.

Different characterization techniques are employed [59,60,61,62,63,64,65,66,67] for assessing the surface morphology, structural, crystallinity, electronic and vibrational characteristics of ${In}_{x}{Ga}_{1-x}N$/Sapphire epifilms. The methods include in situ reflection high-energy electron diffraction (RHEED), scanning electron microscopy (SEM), transmission electron microscopy (TEM), cathodoluminescence (CL), spectroscopic ellipsometry (SE), PL spectra, Hall or van der Pauw measurements, Raman scattering spectroscopy, infrared reflectivity/transmission spectroscopy, Rutherford backscattering, atomic force microscopy (AFM), etc. These studies have identified the formation of In droplets on the ${In}_{x}{Ga}_{1-x}N$ film surface during the growth and found a high electron charge carrier concentration η, with low mobility μ. The growth of defect-free and homogeneous ${In}_{x}{Ga}_{1-x}N$ alloy samples has been challenging. Improvements in the performance of InGaN-based solar cells have been made recently by incorporating strained InGaN/GaN MQWs or SLs as active layer structures [62,63,64]. It has been argued that using ultrathin quantum well layers could mitigate intrinsic defect-related issues.

In the design and fabrication of electronic devices, an accurate value of film thickness, d [68,69,70,71,72], and alloy composition, x [6,7,8], is required. In thicker samples, the SE measurements are performed at energies below $E_{g}$, where the films are transparent and reveal distinct interference fringes. In thin films, however, the SE utilizes a phenomenon of light interference within the film [73,74,75,76,77,78]. Careful examination of changes in the polarization of light reflected from the surface of thin films has allowed extracting epifilm thickness, d, and their optical constants ($\tilde{\varepsilon }\left(E\right)\;or\;\tilde{n}\left(E\right)$). Except for limited results in the far-infrared (FIR) region of semiconductors [79], very few SE studies exist for ${In}_{x}{Ga}_{1-x}N/Sapphireepifilms$ in the NIR → UV energy, E, and/or wavelength, λ, regions. Recently, a transfer matrix method (TMM) was considered as a highly effective approach for studying the transmission $T\left(E\right)$ and reflectivity $R\left(E\right)$ of nanostructured zincblende (zb) ZnCdTe/GaAs epifilms [80,81,82]. Using TMM and simulating x-dependent T(E) and R(E) spectra, we have accurately assessed values of $E_{g}\left(x\right)$ for ultrathin ${In}_{x}{Ga}_{1-x}N/$Sapphire epifilms. A comparison of theoretical results with PL [59,60,61] $E_{g}^{PL}\;$ measurements (cf. Section 3.4) helped estimate the alloy composition, x.

This paper aims to use a semiempirical method (cf. Section 2 and Section 3) for systematically simulating the optical constants ($\tilde{\varepsilon }\left(E\right)\;or\;\tilde{n}\left(E\right))$ of both the binary wz InN, GaN and ternary ${In}_{x}{Ga}_{1-x}N$ alloys in the NIR → UV energy region (i.e., 0.5 eV≤E≤10.5 eV or wavelength 2480 nm≥λ≥118 nm). In Section 2.1, we have briefly outlined the differences between the two major crystalline phases (i.e., the zb and wz structures) of III-N (InN, GaN) materials. The characteristic features of optical constants $\tilde{\varepsilon }\left(E\right)$ $or\;\tilde{n}\left(E\right)$ in semiconductors strongly depend on the critical point (CP) energies of their electronic band structures, $E_{j}\left(\vec{k}\right)$. Due to limited SE measurements on $\tilde{\varepsilon }\left(E\right)$, very few theoretical studies have been performed to comprehend the electronic band structure of ${In}_{x}{Ga}_{1-x}N\;alloys$, until recently [16,17,18,19,20]. By using Perdew–Burke–Ernzerhof (PBE) and Heyd–Scuseria–Ernzerhof (HSE) potentials in density functional theory (DFT), Zhang et al. [18] have reported results of $E_{j}\left(\vec{k}\right)$ and optical dielectric functions of GaN and Ga-rich ${In}_{x}{Ga}_{1-x}N$ alloys. For the wz InN material, Carvalho et al. [16] have performed DFT simulations (cf. Section 2.2) by adopting an AM05 exchange-correlation function to study the $E_{j}\left(\vec{k}\right)$ and its structural properties. Besides identifying the major optical bandgap $E_{0},$ several high-energy transitions ($E_{1\alpha },E_{bi})$ at CPs in the BZ are also perceived [16,17,18]. Considering the band structure of wz InN [16] as an example, we have identified (cf. Section 2.2) the relationships between the valence band splitting caused by crystal-field ${(∆}_{cr})$ and spin-orbit ${(∆}_{so})$ perturbations [8,14]. Following Adachi and including the appropriate inter-band transition energies at CPs [3,4,5] in the BZ, we have developed a semiempirical approach (see Appendix A) using modified dielectric functions (MDFs) to study the optical characteristics of InN, GaN and ${In}_{x}{Ga}_{1-x}N$. Our approach departs from those of Djurišić and Li [83] on two important fronts. First, we have included the correct value of $\;E_{0}$ ~0.7 eV for the wz InN [11,12,13] than the much higher value of 2.247 eV considered in [83]. Secondly, we have systematically included the contributions of high-energy $E_{1\alpha },E_{bi}$ CPs around $\;E_{0}$ from the electronic band structures $E_{j}(\vec{k})$. These changes have enabled us to significantly improve the optical constants $(\tilde{\varepsilon }\left(E\right),\;\tilde{n}\left(E\right)$). The method is used for studying the absorption coefficients $\alpha \left(E\right)$ and normal-incidence reflectivity $R\left(E\right)$ spectra for InN, GaN and ${In}_{x}{Ga}_{1-x}N$ at any arbitrary composition, x, and photon energy, E (cf. Section 2.3). The optical constants of ${In}_{x}{Ga}_{1-x}N$ and sapphire are meticulously integrated into TMM [80,81] for simulating (cf. Section 2.4) the thickness and x-dependent reflectivity $R\left(E\right)$ and transmission $T\left(E\right)$ spectra of nanostructured ${In}_{x}{Ga}_{1-x}N/Sapphire$ epifilms. Careful analyses of $R\left(E\right)$ and $T\left(E\right)$ results have revealed accurate x-dependent shifts of energy gap $E_{g}(x)$ for ${In}_{x}{Ga}_{1-x}N$ (x = 0.5, 0.7) alloys in excellent agreement with the $PLE_{g}^{PL}$ [60,61,62,63,64,65,66] data. The results of numerical computations for optical constants of InN, GaN and ${In}_{x}{Ga}_{1-x}N$ are compared/contrasted in Section 3 with existing experimental data [67,68,69,70,71,72,73,74,75,76,77,78,79,80]. A summary of outcomes with concluding remarks is presented in Section 4.

## 2. Properties of InN, GaN and InGaN

### 2.1. Crystal Structure

**Figure 2:**
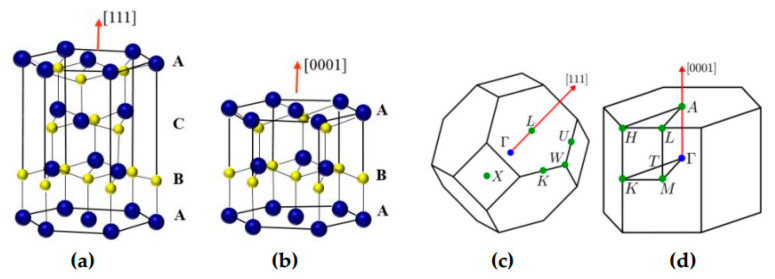
The crystal structure of InN (or GaN) material. Small yellow spheres indicate N atoms while the larger blue spheres represent In or Ga atoms: (**a**) zincblende and (**b**) wurtzite structures. The Brillouin zone of InN or GaN (**c**) for zb and (**d**) wz structures.

InN and GaN materials under ambient conditions occur in a thermodynamically stable (a) wz (hexagonal) crystal structure or α-phase (see Figure 2b) with a space group $P6_{3}mc$ in the Hermann–Mauguin notation $or\;{(C}_{6v}^{4})$ [1] in the Schoenflies [2] notation and (b) a metastable zb (cubic) structure or β-phase (see Figure 2a) with a space group $F\bar{4}3m$ or ($T_{d}^{2}$). The difference between wz and zb structures is the stacking sequence of the close-packed diatomic plane. The α-phase consists of an ABAB stacking sequence of the (0001) close-packed plane while the β-phase comprises an ABCABC stacking sequence of the (111) close-packed plane.

The electronic energy band structures and wave functions are determined mainly from the symmetry of the periodic potentials. Symmetry is expressed by a space group consisting of all the transformations which leave the crystal invariant. The unit cell of the reciprocal lattice is the Brillouin zone (BZ). Figure 2c,d display the BZ of the zb and wz structures. Table 1 lists the basic structural and electronic properties of the wz and zb InN and GaN materials.

### 2.2. Band Structure of InN, GaN and ${In}_{x}{Ga}_{1-x}N$ Ternary Alloys

**Figure 3:**
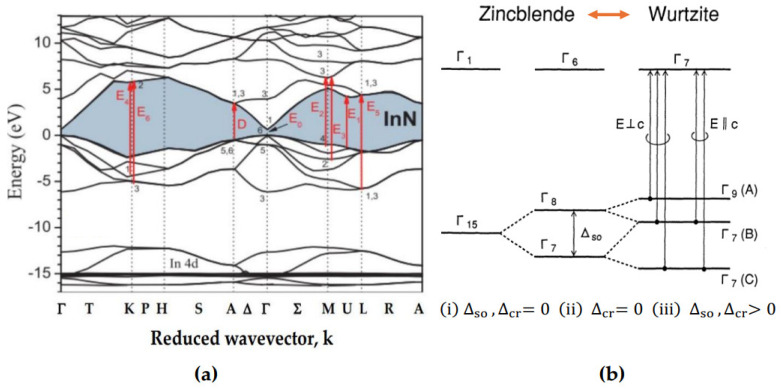

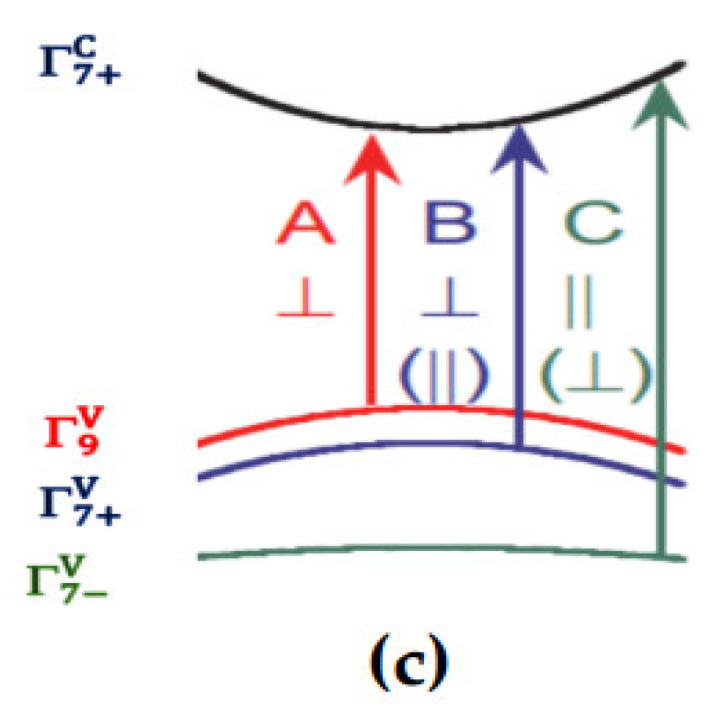
(**a**) Calculated DFT band structure of wz InN with AM05 exchange-correlation function without spin-orbit interaction [16]. Red vertical arrows indicate inter-band transitions in the E⏊c configuration. (**b**) Relationship [3,4,5] between crystal-field ${∆}_{cr}$ and spin-orbit ${∆}_{so}$ perturbations in zb and wz lattices. (**c**) Simplified scheme of A, B and C transitions due to ${∆}_{cr}\;$ and $\;{∆}_{so}$.

In Figure 3a, we have displayed the electronic energy band structure $E_{j}\left(\vec{k}\right)$ for the wz InN material as simulated by Carvalho et al. [16]. The authors adopted a DFT methodology in the framework of an AM05 exchange-correlation functional scheme [16]. The calculated lattice parameters $a_{0}$ = 3.549 (Å) and $c_{0}$ = 5.736 (Å) are found in reasonably good agreement (see Table 1) with the experimental values 3.538 (Å) and 5.704 (Å), respectively, reported by Paszkowicz et al. [19].

The red vertical arrows in Figure 3a indicate the locations of several inter-band transitions at CPs in the BZ. Besides identifying the main optical transition $E_{0},$ several high-energy transitions ($E_{1\alpha },E_{bi})$ are also perceived [16]. In the E⏊c polarization, these CPs have played valuable roles in the development of empirical modified dielectric functions for simulating the optical constants (see Appendix A) of wz binary InN, GaN and ternary ${In}_{x}{Ga}_{1-x}N$ alloys [3,4,5].

#### 2.2.1. Optical Transitions in InN, GaN and ${In}_{x}{Ga}_{1-x}N$ Alloys

At room temperature, the InN (GaN and/or ${In}_{x}{Ga}_{1-x}N$ alloys) crystallizes in the wz structure (with space group $C_{6v}^{4}$). The hexagonal InN (GaN) material has a direct energy bandgap $E_{0}:\Gamma_{6}\to \Gamma_{1}$ of ~0.7 eV (~3.42 eV) [14,15,16,17,18] which means that the conduction band minimum and the valence band maximum are in the center of BZ at the Γ point (see Figure 3a). The Bloch wave functions of the conduction band and valence band are described by the s-states and p-states, respectively. Figure 3b reveals the relationship between the valence band splitting in the zb and wz structures caused by crystal-field ${∆}_{cr}$ and spin-orbit ${∆}_{so}$ perturbations [3,4,5]. In the wz structures, the crystal field splitting appears due to the structural anisotropy. The conduction and valence band structures for the wz material are illustrated in Figure 3b. As seen in Figure 3b, the triplet states of zb ($\Gamma_{15}$) split up into $\Gamma_{9}\left(A\right)$, $\Gamma_{7}\left(B\right)\;and\;\Gamma_{7}\left(C\right)$ valence bands for AlN with the combined effects of ${∆}_{so}<0,\;{∆}_{cr}>0.$ The values of ${∆}_{so}$, ${∆}_{cr}>0$ for α-phase InN (GaN) are reported in Table 1. Consequently, one expects the absorption edge of ${In}_{x}{Ga}_{1-x}N$ alloy to exhibit three excitonic structures. These structures become sharper at lower temperatures. In the wz material, the $\Gamma_{7}^{c}$ conduction band exhibits an s-like symmetry, while $\Gamma_{9}^{v}\left(A\right),$ $\Gamma_{7}^{v}\left(B\right)$ and $\Gamma_{7}^{v}\left(C\right)$ valence bands (see Figure 3b) show p-like behavior.

Again, the polarization vectors E⏊c and E||c of the space group $C_{6v}^{4}$ belong to $\Gamma_{5}$ and $\Gamma_{1}$ symmetries [3,4]. From group theoretical arguments, a direct product of $\Gamma_{7}\times$ $\Gamma_{7}$ = $\Gamma_{1}$ + $\Gamma_{2}$ + $\Gamma_{5}$ contains the representations of both E⏊c and E||c polarizations, while the product $\Gamma_{9}\;\times$ $\Gamma_{7}$ = $\Gamma_{5}$ + $\Gamma_{6}$ holds only the representation of E⏊c polarization [3,4], which simply means that, in the E||c polarization, the optical transitions at the Γ point in the BZ are forbidden among the A valence and conduction bands, while for the Ec all the optical transitions (see Figure 3b,c) are allowed. In the SE optical $\tilde{\varepsilon }\left(E\right)$ spectra of the wz InN material, the two dominant features noticed for the E⏊c polarization are located at energies near 5.35 eV and 6.29 eV, respectively. A secondary weak structure is also evident at an energy close to 6.75 eV [14,15,16,17,18]. In the wz InN (or GaN) material, these characteristics are seen in an energy region higher than that of the $E_{0}$ value.

One usually categorizes these traits as $E_{1\alpha }$ (with α≡A,B,C) energy bandgaps. The assignment of such structures was first proposed by Cardona [84]. These energy attributes ($E_{1A}$, $E_{1B}$, $E_{1C}$) in the wz structures correspond to the $E_{1}$ and $E_{1}+{∆}_{1}$ transitions in the zb materials. For the E||c polarization, only an $E_{1C}$ peak is observed, while the $E_{1A}$ and $E_{1C}$ structures are noticed in the E⏊c polarization (see Figure 3b,c). Due to the spin-orbit interaction, the $E_{1A}$ energy splits up into two states denoted as $E_{1A}$ and $E_{1B}$. The $E_{1A}$ peak could be related to a transition along the Γ → A direction of the BZ, while the $E_{1B}$ structure might be associated with the transition along the M-U-L direction or near the M point in the BZ [16]. One must also note that the energy peaks $E_{1A}$ and $E_{1B}$ are forbidden in the E||c polarization, i.e., only the $E_{1C}$ peak appears in the E||c polarization. This $E_{1C}$ energy structure is suggested to originate from the transitions at the U point in the BZ [3]. Thus, in our construction of the MDF formalisms for wz InN (and GaN) material, we have considered (see Appendix A) $E_{1\alpha }$(α≡A,B,C) gaps from the electronic band structure $E_{j}\left(\vec{k}\right)$ [16] (cf. Figure 3a). These $E_{1A},$ $E_{1B}$ and $E_{1C}$ energies are observed in SE measurements near ~5, 6 and 7 eV (and ~6, 8 and 9 eV), respectively [67,68,69,70,71,72,73,74,75,76,77,78,79,80] in the E⏊c polarization.

#### 2.2.2. Photoluminescence Measurements in ${In}_{x}{Ga}_{1-x}N$ Alloys

**Figure 4:**
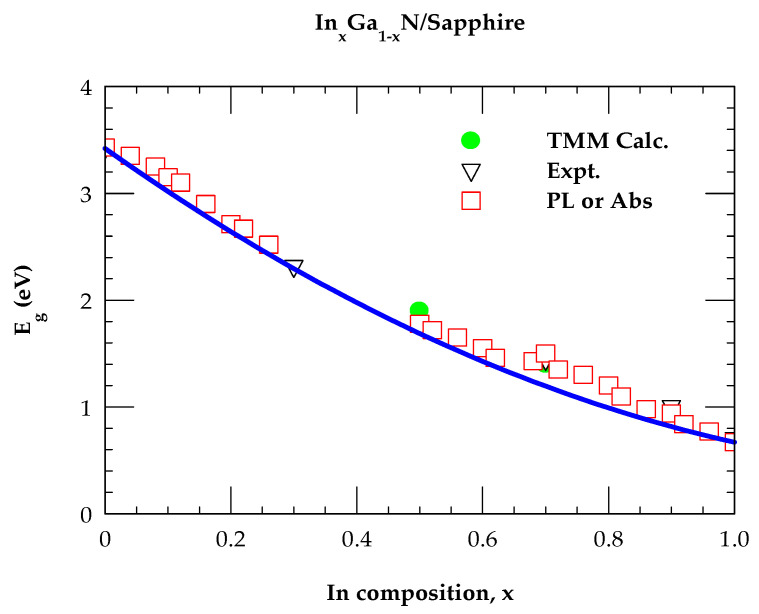
Comparison of the composition-dependent PL data of $E_{g}$(x) for In_x_Ga_1−x_N/Sapphire. The red squares and black inverted triangles are the experimental data that are contrasted against the calculated results (blue line) obtained by using Equation (1). Solid green spheres represent the bandgap value estimated from the $R\left(E\right)$ and $T\left(E\right)$ spectra using TMM.

Several In-rich In_x_Ga_1−x_N/Sapphire films of thickness d (≡0.2–1.2 μm) have been grown by MOCVD and MBE [8,9,10,11,12,13] by including an AlN buffer layer of a few nanometers in thickness. These epifilms are used in different optoelectronic devices [1,2,3,4,5,6,7,8,9,10]. The PL study has exhibited a decrease in bandgap $E_{g}$ $(\approx E_{0})\;$ from ~3.42 eV of GaN down to ~1.4 eV for In_0.7_Ga_0.3_N alloy. Many optical measurements have revealed a strong dependence of the fundamental bandgap on the In composition, x. Better-quality wz InN/Sapphire samples have exhibited intense PL spectra at energies around ~0.7 eV [11,12,13]. Along with the PL spectra, a clear absorption edge and photomodulated reflectance transition have also been observed [15], confirming the fundamental bandgap of InN near ~0.7 eV. In Figure 4, we have reported a comparison between the calculated composition-dependent $E_{g}$(x) with the PL data. The solid blue line shows the results based on a standard quadratic equation [85]:

Eg(x) = 3.42 (1 − x) + 0.67 (x) − b × (1 − x),(1)

which provided a good fit with the choice of $b\left(=1.43eV\right)$ as a bowing parameter [85].

Again, Figure 4 has not only offered a good accord of $E_{g}$(x) with PL measurements but also revealed accurate bandgaps of In_x_Ga_1−x_N/Sapphire for x = 0.5 and 0.7 that we obtained from our meticulous studies of $R\left(E\right)$/$T\left(E\right)\;spectra$ using the transfer matrix method (cf. Section 3.4).

### 2.3. Model Dielectric Functions of InN, GaN and ${In}_{x}{Ga}_{1-x}N$

In the framework of a semiempirical formalism [3,4,5], we have constructed the modified model dielectric functions for the binary wz InN, GaN and ternary ${In}_{x}{Ga}_{1-x}N$ materials. The MDFs are carefully obtained following the procedure succinctly outlined in Appendix A. Adachi offers a complete description of the methodology elsewhere [5]. In this approach, the CP energies (viz., $E_{0}$, $E_{0\alpha }$, $E_{1\alpha }\;$(with α≡A,B,C), and $E_{abi}$) have provided the major contributions to the dispersion mechanisms for comprehending the optical $(\tilde{\varepsilon }\left(E\right)$ or $\tilde{n}\left(E\right)$) constants. The calculated refractive indices $\tilde{n}\left(E\right)$ are meticulously incorporated in the multi-layer TMM [81] (cf. Section 2.4) approach for studying the thickness-dependent R(E) and T(E) spectra of nanostructured ${In}_{x}{Ga}_{1-x}N$/Sapphire epiflms (cf. Section 3.1,Section 3.2,Section 3.3,Section 3.4). For x = 0.5 and 0.7, the values obtained for the energy bandgaps $E_{g}$(x) of In_x_Ga_1−x_N/Sapphire from TMM calculations (cf. Section 3.4) of $R\left(E\right)\;$ and $T\left(E\right)$ spectra agreed reasonably well with the PL and/or the absorption edge measurements [85].

#### Model Dielectric Function of Sapphire

Sapphire (${\alpha -Al}_{2}O_{3}$) is an outstanding insulator having exceptional physical characteristics, including hardness, high thermal conductivity, high optical transparency, etc. The material exhibits considerable mechanical strength and the ability to withstand high temperatures. This makes ${\alpha -Al}_{2}O_{3}$ an ideal substrate for epitaxial growth of ultrathin semiconducting films including SiC and III-Ns. The nanostructured epifilms grown on sapphire are frequently used in various optoelectronic device applications [1,2,3,4,5,6,7,8,9,10]. To assess the optical characteristics of In_x_Ga_1−x_N/${\alpha -Al}_{2}O_{3}$ epifilms using TMM, we have obtained the dielectric function of the sapphire material by using a Sellmeier equation [6,7]:

(2) $$\varepsilon_{Sapphire}=1+\frac{A\lambda^{2}}{\lambda^{2}-B}+\frac{C\lambda^{2}}{\lambda^{2}-D}+\frac{E\lambda^{2}}{\lambda^{2}-F},$$

with the values of coefficients A = 1.0237980, B = 0.0037759, C = 1.0582640, D = 0.0122544, E = 5.2807920, F = 321.36160.

### 2.4. Transfer Matrix Method for Multi-Layered Structure

Interference methodologies are frequently used for estimating the film thickness in layered crystalline structures [67,68,69,70,71,72,73,74,75,76,77,78,79,80]. Theoretically, the summation of multiplying the reflected and transmitted waves in a multi-layered arrangement of materials is a cumbersome process. Fortunately, a well-known TMM [80,81] approach can offer an elegant solution to this complex problem. Recently, some authors have developed elaborate numerical and analytical methods using TMM for analyzing the R(E) and/or transmission T(E) spectra [86]. Comparison of the simulated reflectivity spectra using TMM in the near-IR (NIR) to UV–Vis energy region with experimental data has offered an accurate assessment of the film thickness, d, for ultrathin zb ${Zn}_{x}{Cd}_{1-x}Se(Te)$/GaAs (001) samples [86]. Alternatively, we have used the TMM approach for numerically simulating the R(E)/T(E) spectra for wz ${In}_{x}{Ga}_{1-x}N/Sapphire$ epilayers of different composition, x, and thickness, d. Assessment of the calculated energy bandgap $E_{g}$ values with PL measurements $E_{g}^{PL}$ has supported the accuracy of TMM in studying the optical spectra of ultrathin films in the NIR → UV–Vis region. We strongly feel that this approach can be easily extended to other technologically important nanostructured materials.

In TMM, we have assumed stacking j-number of layers, each with thickness $d_{j}$ and refractive index ${\tilde{n}}_{j}\left(E\right)$ to form a multi-layer structure. Each jth layer in such a structure is characterized by a 2 × 2 matrix [81]:

(3) $$M_{j}=\left(\begin{array}{cc} cos(k_{0}\;{\tilde{n}}_{j}d_{j}) & \frac{i}{{\tilde{n}}_{j}}sin(k_{0}\;{\tilde{n}}_{j}d_{j})\\ i{\tilde{n}}_{j}sin(k_{0}\;{\tilde{n}}_{j}d_{j}) & cos(k_{0}\;{\tilde{n}}_{j}d_{j}) \end{array}\right),$$

where k0(=2πλ) is the wavevector of the monochromatic light; the matrix M of the multi-layer system is a product of appropriate individual layer matrices [81]:

(4) $$M=\left(\begin{array}{cc} m_{11} & m_{12}\\ m_{21} & m_{22} \end{array}\right)=M_{1}M_{2}\ldots ..M_{m-1}$$

An important feature of the matrix M is that its determinant is unity. Applying this property to a periodic system, the reflection ($\tilde{r}$) and transmission ($\tilde{t}$) coefficient can be evaluated by using

(5) $$\left(\begin{array}{c} 1+\tilde{r}\\ {\tilde{n}}_{0}(1-\tilde{r}) \end{array}\right)=M\left(\begin{array}{c} \tilde{t}\\ {\tilde{n}}_{m}\tilde{t} \end{array}\right),$$

where different refractive indices ${\tilde{n}}_{0}$ and ${\tilde{n}}_{m}$ are considered on either side of the multi-layer system. Following Heavens [87], the $\tilde{r}$ and $\tilde{t}$ are expressed as:

(6) $$\tilde{r}=\frac{m_{11}{\tilde{n}}_{0}-m_{21}+m_{12}{\tilde{n}}_{0}{\tilde{n}}_{m}-m_{22}{\tilde{n}}_{0}{\tilde{n}}_{m}}{m_{21}+m_{11}{\tilde{n}}_{0}+m_{12}{\tilde{n}}_{0}{\tilde{n}}_{m}+m_{22}{\tilde{n}}_{m}},$$

(7) $$\tilde{t}=\frac{2{\tilde{n}}_{0}}{m_{21}+m_{11}{\tilde{n}}_{0}+m_{12}{\tilde{n}}_{0}{\tilde{n}}_{m}+m_{22}{\tilde{n}}_{m}}.$$

For each film of the multi-layer system, one needs to have the transfer matrices multiplied (cf. Equation (4)) and then substituted into Equations (6) and (7) to obtain the appropriate components $m_{ab.}$ For a single-layer epifilm structure, once $\tilde{r}$ and $\tilde{t}$ are achieved, it is straightforward to simulate the energy-dependent R(E) and $T\left(E\right)$ by using:

(8) $$R\left(E\right)=\left|{\tilde{r}}^{2}\right|,$$

(9) TE=n~m|r~2|n~0.

At near normal incidence ($\theta_{i}$ = 0), our results of R(E) and $T\left(E\right)$ spectra for ultrathin wz ${In}_{x}{Ga}_{1-x}N/Sapphire$ provided accurate assessment (cf. Section 3.4) of bandgaps of different In compositions, x, and thicknesses, d, which validates the importance of TMM. We strongly feel that it can be used for other technologically important materials.

## 3. Numerical Computations, Results and Discussion

Different theoretical models exist for studying the complex dielectric functions of wz InN and GaN materials [3]. Despite the efforts made in studying $E_{j}\left(\vec{k}\right)$, reports on the optical characteristics are rather scarce. Significant variations exist between the simulated and experimental values of $\tilde{\varepsilon }\left(E\right)$. While the calculated positions of sharper peaks in $\tilde{\varepsilon }\left(E\right)\;$ agreed with the absorption spectrum, the observed Gaussian-shaped broadening of energy curves is not well understood. To comprehend the Gaussian shape broadening, Kim et al. [88] employed an empirical model and replaced the CP damping constant Γ with the energy-dependent damping Γ′(E).

### 3.1. Modified Dielectric Function Parameters

We have adopted a semiempirical method [3,4,5] (cf. Section 2.3) for constructing the MDFs for binary wz InN and GaN materials by incorporating appropriate CP energies for simulating $\tilde{\varepsilon }\left(E\right)$ ($\tilde{n}\left(E\right)$) in the E⏊c polarization. Integrating the optical constants of different ultrathin layers in TMM can help facilitate the accurate design of the LDH-based device structures for optoelectronic applications. Following Adachi’s methodology [3,4,5], we have carefully included appropriate contributions (see Appendix A and Table 2) of different CPs to develop the MDFs.

The model parameters reported in Table 2 for binary wz GaN and InN materials are extended to the ternary ${In}_{x}{Ga}_{1-x}N$ alloys. The simulated results of optical constants $\tilde{\varepsilon }\left(E\right),\tilde{n}\left(E\right),$ including the R(E) and $\;\alpha \left(E\right)$ coefficients (cf. Section 3.2 and Section 3.3), have agreed reasonably well with the existing experimental data [67,68,69,70,71,72,73,74,75,76,77,78,79].

#### Dielectric-Related Optical Constants

The components of complex refractive index $\tilde{n}\left(E\right)\left[\equiv n\left(E\right)+i\kappa \left(E\right)\right]$ are directly related to the elements of the complex dielectric function $\tilde{\varepsilon }\left(E\right)\left[\equiv \varepsilon_{1}\left(E\right)+i\varepsilon_{2}\left(E\right)\right]$ by [3]:

(10) $$n\left(E\right)={\left(\frac{[{\varepsilon_{1}\left(E\right)}^{2}+{\varepsilon_{2}\left(E\right)}^{2}{]}^{\frac{1}{2}}+\varepsilon_{1}\left(E\right)}{2}\right)}^{1/2},\;\;\kappa \left(E\right)={\left(\frac{[{\varepsilon_{1}\left(E\right)}^{2}+{\varepsilon_{2}\left(E\right)}^{2}{]}^{\frac{1}{2}}-\varepsilon_{1}\left(E\right)}{2}\right)}^{1/2}.$$

The optical absorption coefficient $\alpha \left(E\right)$, and the normal-incidence reflectivity $R\left(E\right)$ spectra can be expressed using $n\left(E\right)$ and $\kappa \left(E\right)\;$ as [3]:

(11) $$\alpha \left(E\right)=\frac{4\pi }{\lambda }\kappa \left(E\right),$$

(12) $$R\left(E\right)=\frac{{\left[n\left(E\right)-1\right]}^{2}+{\kappa \left(E\right)}^{2}}{{\left[n\left(E\right)+1\right]}^{2}+{\kappa \left(E\right)}^{2}},$$

where λ is the wavelength of light in the vacuum.

### 3.2. Optical Constants of Binary Materials

**Figure 5:**
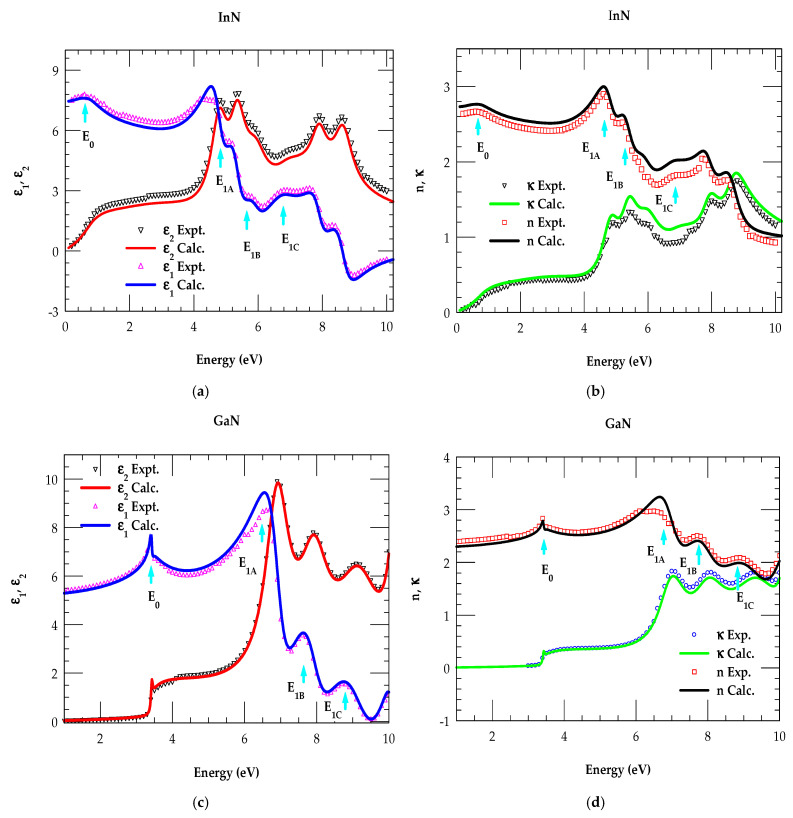
Comparison of the simulated optical constants (**a**) $\varepsilon_{1}\left(E\right),\varepsilon_{2}\left(E\right)\;$ and (**b**) $n\left(E\right)$, $\kappa \left(E\right)\;$ with the experimental datafor wz InN. Same key for (**c**) $\varepsilon_{1}\left(E\right),\varepsilon_{2}\left(E\right)$ and (**d**) $n\left(E\right)$, $\kappa \left(E\right)$ of the wz GaN.

Figure 5a,b show, respectively, the real and imaginary parts of the dielectric function ($\varepsilon_{1}\left(E\right),\varepsilon_{2}\left(E\right))$ and refractive index $(n\left(E\right)$, $\kappa \left(E\right))$ for wz InN material as a function of energy E. Different colored symbols represent the experimental data acquired from the literature [68,69,70,71,72,73,74,75].

In contrast, solid-colored lines are our results of the MDF calculations. Similar results are reported for the wz GaN material in Figure 5c,d, respectively. A comparison of MDF simulations uses solid-colored lines and experimental data [68,69,70,71,72,73,74,75] indicated by symbols. Figure 5a–d show excellent agreement between the theoretical and experimental results. Unlike others [83] our MDF simulations revealed the major CP transition energies $(E_{0}$ and $E_{1A}$, $E_{1B}$, $E_{1C}\;)\;of$ the InN and GaN materials. The locations of these transition energies, indicated by sky-blue vertical arrows, are in excellent agreement with the values reported in the electronic energy band structure of InN [16] and GaN [18].

#### Reflectivity and Absorption Coefficients of Binary Materials

**Figure 6:**
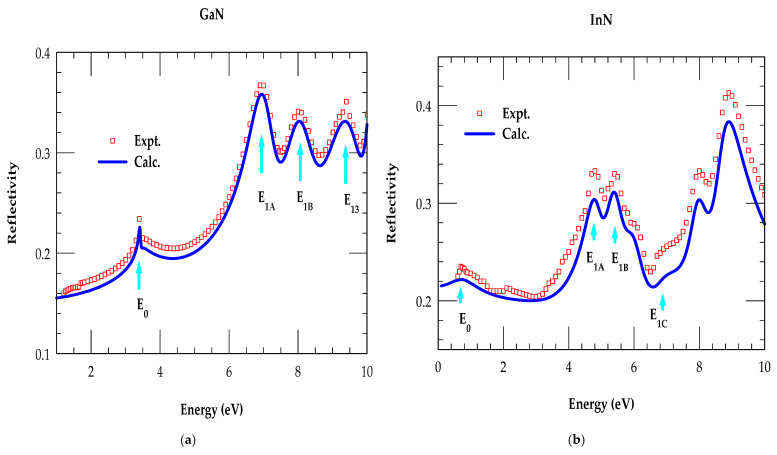

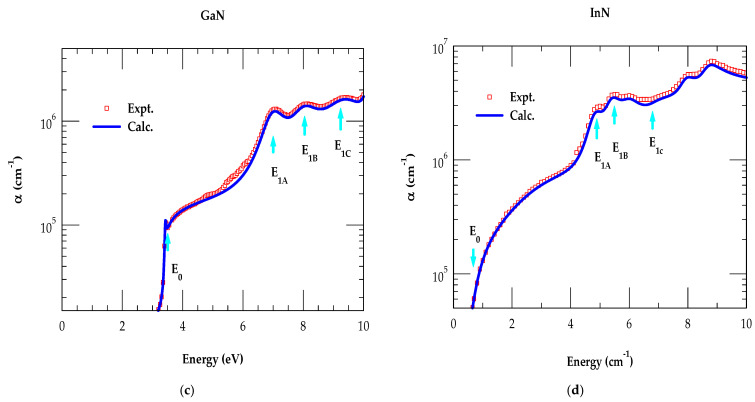
Comparison of simulated Reflectivity $R\left(E\right)$ spectra with experimental data for wz GaN (**a**) and wz InN (**b**) materials. The same key for the absorption coefficient of $\alpha \left(E\right)$: GaN (**c**), InN (**d**).

Earlier, measurements were performed on hexagonal InN [68,69,70,71,72,73,74] and GaN [75,76,77,78,79] materials for assessing their fundamental reflectivity $R\left(E\right)$ and absorption $\alpha \left(E\right)$ spectra. More recently, accurate simulations with improved experimental measurements for InN [68,69,70,71,72,73,74] have also been reported. Using Equations (11) and (12), we have performed MDF calculations of $\alpha \left(E\right)$ and $R\left(E\right)$ spectra as a function of energy E. Figure 6a,b show results of $R\left(E\right)$ for the wz GaN and InN materials, respectively, where calculations indicated by solid, blue-colored lines are compared with experimental results, represented by red-colored open squares. Figure 6c,d show an excellent comparison between MDF simulations and experimental data [68,69,70,71,72,73,74,75,76,77,78,79] for the absorption $\alpha \left(E\right)$ spectra of GaN and InN, respectively. Like the real and imaginary parts of optical constants ${(\varepsilon }_{1}\left(E\right),\varepsilon_{2}\left(E\right)$; $n\left(E\right),\;\kappa \left(E\right))$, our results for $\alpha \left(E\right)$ and $R\left(E\right)$ spectra have clearly shown the distinct CP energy transitions $E_{0}$, $E_{1A}$, $E_{1B}$ and $E_{1C}.$ Again, these energy values of inter-band transitions indicated by the sky-blue-colored vertical arrows are in good agreement with the energy band structure calculations [16,18].

### 3.3. Optical Constants of In_x_Ga_1-x_N Ternary Alloys

**Figure 7:**
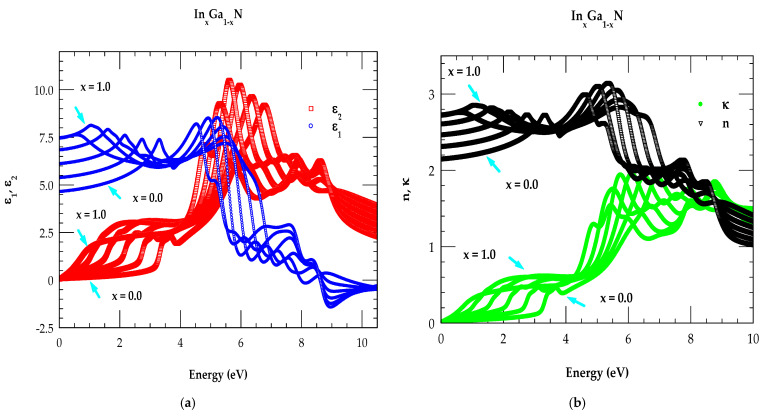

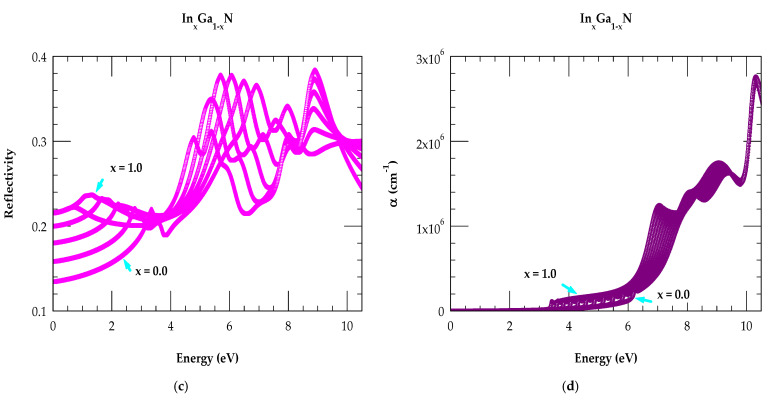
(**a**) Simulation of E-dependent $\varepsilon_{1}$(E), $\varepsilon_{2}$(E) for the In_x_Ga_1−x_N alloys with an increment of composition x = 0.2. (**b**) Simulation of E-dependent n(E), κ(E) for the In_x_Ga_1−x_N alloys with an increment of In composition x = 0.2. (**c**) same as (**b**) but for Reflectivity. (**d**) same as (**b**) but for absorption coefficient.

Table 2 summarizes the parameters obtained in the MDF scheme for the wz GaN and InN materials. By assuming a linear dependence on the composition of these parameters, one can calculate optical response in In_x_Ga_1−x_N ternary alloys with any arbitrary composition, x, and photon energy, E. In Figure 7a–d we have displayed our simulated MDF results for the real and imaginary parts of the dielectric constants ($\varepsilon_{1}\left(E\right),\varepsilon_{2}\left(E\right)$), refractive indices $(n\left(E\right)$, $\kappa \left(E\right)$), reflectivity $R\left(E\right)$ and absorption coefficient $\;\alpha \left(E\right)$, respectively.

The MDF simulations reported using different colors are performed with an increment of 0.2 for the In composition, x. From Figure 7a,d, it can be noticed that, as x increases, the spectral features shift towards the low-energy side. These results are in accordance with the low-energy shifts of the $E_{0}$, $E_{1A}$, $E_{1B}$ and $E_{1C}$ inter-band transitions [61,62,63,64,85] with increasing x.

### 3.4. Thickness- and Composition-Dependent Reflectivity and Transmission Spectra

By carefully incorporating the optical constants of both the ${\alpha -Al}_{2}O_{3}$ substrate and In_x_Ga_1−x_N epifilms in the TMM approach, one might be able to systematically simulate the results of the reflectivity R(E) and transmission T(E) spectra for any composition, x, and layer thickness, d, in the In_x_Ga_1−x_N/Sapphire epilayers.

**Figure 8:**
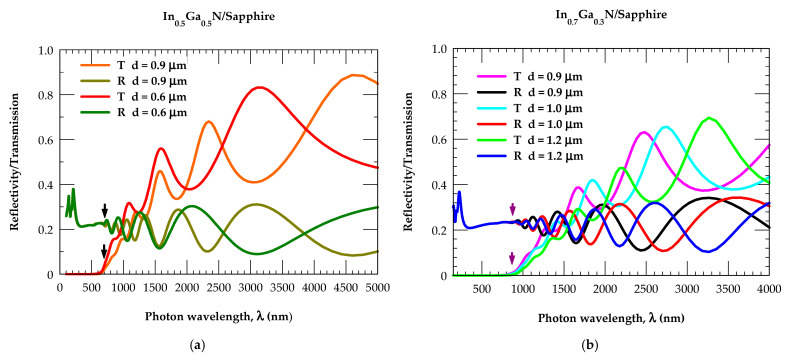
(**a**) Transfer-matrix-based simulated spectra of reflectance R(E) (red-, orange-colored line) and transmission T(E) (green-, bottle-green-colored line) for the 0.9 μm and 0.6 μm thick In_0.5_Ga_0.5_N epifilm grown on the sapphire substrate. The black-colored vertical arrows drawn near 640 nm show a bandgap of ~1.94 eV, in excellent agreement with the existing PL data from the literature [61,62,63,64,85]. (**b**) The same key as for Figure 7a but for In_0.7_Ga_0.3_N/Sapphire epifilms of several thicknesses d, using different colored lines for reflectivity and transmission spectra. Black-colored vertical arrows drawn near 860 nm show an appropriate shift of the bandgap ~1.44 eV, in excellent agreement with the existing PL data from the literature [61,62,63,64,85].

Careful analyses of the spectral results would be valuable to assess the composition-dependent energy bandgaps. In Figure 8a we have displayed our results by using different colored lines for the R(E) and T(E) spectra of In_0.5_Ga_0.5_N epilayer with thickness d = 0.6 μm and 0.9 μm. Vertical, black-colored arrows drawn near 640 nm (≡1.94 eV) in the reflectivity and transmission spectra clearly show the simulated energy bandgap ($E_{g}^{TMM})\;by\;the\;TMM$ approach to be in excellent agreement with the photoluminescence ($E_{g}^{PL}$) measurements (see Figure 4) for In_0.5_Ga_0.5_N/Sapphire epilayers [85]. Similar calculations (see Figure 8b) are also performed for a different In composition (In_0.7_Ga_0.3_N/Sapphire epilayers) with thickness d = 0.9 μm, 1.0 μm and 1.2 μm. The vertical, black-colored arrows shown near 860 nm have revealed the accurate x-dependent $E_{g}^{TMM}$ energy bandgap for the alloy to be in good agreement with the PL experimental [85] result, $E_{g}^{PL}$ near ~1.44 eV.

## 4. Concluding Remarks

Since the discovery of a lower energy bandgap $E_{g}\sim 0.7\;eV$ for the hexagonal InN in 2003, ultrathin ${In}_{x}{Ga}_{1-x}N/Sapphireepifilms$ have attracted significant attention among the scientific and engineering community. While InN material with a narrow bandgap and high electron mobility is considered promising for optoelectronics, ${In}_{x}{Ga}_{1-x}N$ epifilms have already been used in many commercial devices including LDs, LEDs in TVs, LCD monitors, mobile displays, solid-state lighting, etc. Several experimental studies identified the presence of deep defects in ${In}_{x}{Ga}_{1-x}N$ layers. Many researchers have alluded to point defects as a source of efficiency reduction in optoelectronic devices [7,8,9]. Identifying the microscopic origin of defects in such an alloy is complicated as the defect levels are likely to exhibit different sensitivities to changes in the In concentration, bandgap and band edges and distribution of In and Ga cations. Establishing how charge-state transition levels evolve in an ${In}_{x}{Ga}_{1-x}N$ alloy as a function of x is essential. The positions of impurity states within $\;E_{g}$ determine optical transitions in photoionization spectra involving defect levels and band edges. We did not consider these effects of defects in our simulations of optical properties for ${In}_{x}{Ga}_{1-x}N$. Several theorists have used computationally intensive methods to study the electronic band structures $E_{j}\left(\vec{k}\right)$ of binary materials and subsequently derive their optical properties [16,18]. Despite significant efforts in the growth of ${In}_{x}{Ga}_{1-x}N$/Sapphire epilayers, no x- and d-dependent optical studies exist for the ultrathin films. Following Adachi [5] and by meticulously including the inter-band transitions ($E_{0},E_{1\alpha },E_{bi}$) at critical points in the BZ, we have developed a semiempirical MDF approach for simulating the optical characteristics of wz InN, GaN and ${In}_{x}{Ga}_{1-x}N$ alloys. Our method differs from that of Djurišić and Li [83] on two important fronts. First, we have included the correct lower value of $E_{0}$ ~0.7 eV for the wz InN [11,12,13] material than the much higher value of 2.247 eV considered in [83]. Secondly, we have systematically integrated the contributions of high-energy $E_{1\alpha },E_{bi}$ CPs around $\;E_{0}$ from the electronic energy band structures $E_{j}\left(\vec{k}\right)$[16,18]. These changes have enabled us to significantly improve the optical constants ($\tilde{\varepsilon }\left(E\right),\;\tilde{n}\left(E\right)$). The method has been used to study the absorption coefficients $\alpha \left(E\right)$, and normal-incidence reflectivity $R\left(E\right)$ for InN, GaN and ${In}_{x}{Ga}_{1-x}N$ at any arbitrary composition, x, and photon energy, E. Results of our numerical computations for optical constants for InN, GaN and ${In}_{x}{Ga}_{1-x}N$ are compared/contrasted with the existing experimental data [61,62,63,64,85], achieving excellent agreement. The refractive indices of ${In}_{x}{Ga}_{1-x}N$ alloys and sapphire materials are methodically integrated into the TMM [81] formalism for simulating the thickness and composition-dependent reflectivity $R\left(E\right)$ and transmission $T\left(E\right)$ spectra of the nanostructured ${In}_{x}{Ga}_{1-x}N/Sapphire$ epifilms. Careful analyses of the $R\left(E\right)$ and $T\left(E\right)$ results have revealed accurate x-dependent shifts of the energy bandgap $E_{g}(x)$ for ${In}_{x}{Ga}_{1-x}N$ (x = 0.5, 0.7) alloys in excellent agreement with the $PLE_{g}^{PL}$ [61,62,63,64,85] measurements. We strongly feel that this approach can be easily extended to many other technologically important ternary alloys for assessing the alloy composition values, x.

## Figures and Tables

**Table 1:** Basic structural and electronic properties of the wz and zb InN and GaN materials.

Parameter	wz InN	Others ^(a)^	zb InN	Others ^(a)^	wz GaN	Others ^(a)^	zb GaN	Others ^(a)^
$E_{0}(eV)$	0.70	0.72, 0.69	0.56	0.54, 0.53	3.43	3.435	3.07	
a(Å)	3.549	3.49, 3.533	5.01	5.169, 4.98	3.189	3.156, 3.242	4.49	4.461, 4.582
c(Å)	5.736	5.61, 5.693			5.185	5.280, 5.145		
Thermal stability (°C)	630				800–900			
Saturated velocity (cm/s)	3.5 × 10^7^				2.0 × 10^7^			
Spontan. pol const. C/m^2^	1.026				1.312			
*K* (W·m^−1^·K^−1^)	120	80, 142			227	130, 253		
μ (cm^2^/Vs)	3000	2500–2700			1000	900–1100		
$m_{⏊}^{*}(m_{0})$	0.071		0.054		0.212		0.193	
$m_{\left|\right|}^{*}(m_{0})$	0.067				0.190			
${∆}_{cr}(meV)$	67	40, 35.6			9.2	17, 28.5		
${∆}_{so}(meV)$	13	5			18.9	10, 15.9		

^(a)^ Refs. [8,9,10,11,12,13,14,15,16,17,18,19].

**Table 2:** Following Adachi [5] and others [47], we have reported the MDF parameters for calculating the optical constants of the wz GaN, InN and In_x_Ga_1−x_N materials.

Parameter	GaN	InN	In_x_Ga_1−x_N
$\varepsilon_{\infty }$	0.426	1.50	0.426 + 1.074x
$A({eV}^{1.5})$	41.251	3.000	41.251 − 38.251x
$\Gamma_{0}(eV)$	0.287	0.300	0.287 + 0.013x
$\alpha_{0}$	1.241	1.241	1.241 + 0.0x
$E_{0}(eV)$	3.42	0.70	3.42 − 2.72x
$B_{1A}(eV)$	0.778	1.10	0.778 + 0.322x
$B_{1B}(eV)$	0.103	0.700	0.103 + 0.597x
$B_{1C}(eV)$	0.920	0.550	0.92 − 0.37x
$B_{1A}^{X}(eV)$	2.042	1.042	2.042 − 1.0x
$B_{1B}^{X}(eV)$	1.024	0.624	1.024 − 0.4x
$B_{1C}^{X}(eV)$	1.997	1.400	1.997 − 0.597x
$\Gamma_{1A}(eV)$	0.743	0.300	0.743 − 0.443x
$\Gamma_{1B}(eV)$	0.428	0.380	0.428 − 0.048x
$\Gamma_{1C}(eV)$	0.440	0.320	0.440 − 0.12x
$G_{1A}^{2D}(eV)$	0.0003	0.0003	0.0003 + 0.0x
$G_{1B}^{2D}(eV)$	0.356	0.356	0.356 + 0.0x
$G_{1C}^{2D}(eV)$	1.962	1.962	1.962 + 0.0x
$E_{1A}(eV)$	6.010	5.353	6.010 − 0.657x
$E_{1B}(eV)$	8.182	6.290	8.182 − 1.892x
$E_{1C}(eV)$	8.761	6.750	8.761 − 2.011x
$A_{0}^{ex}(eV)$	0.249	0.010	0.249 − 0.239x
$G_{0}^{3D}(eV)$	0.030	0.020	0.030 − 0.01x
$E_{ab1}(eV)$	3.900	3.900	3.900 + 0.0x
$S_{ab1}(eV)$	0.000	29.00	0.000 + 29.00x
$\Gamma_{ab1}(eV)$	5.200	5.200	5.200 + 0.0x
$E_{ab2}(eV)$	7.900	7.900	7.900 + 0.0x
$S_{ab2}(eV)$	0.000	10.00	0.00 + 10.00x
$\Gamma_{ab2}(eV)$	0.600	0.600	0.600 + 0.0x
$E_{ab3}(eV)$	8.650	8.650	8.650 + 0.0x
$S_{ab3}(eV)$	0.000	17.00	0.00 + 17.00x
$\Gamma_{ab3}(eV)$	0.700	0.700	0.700 + 0.0x

## Data Availability

The data that support the findings of this study will be available from the corresponding author upon reasonable request.

## Appendix A

Kramers–Krönig (KK) relationships in materials are generally linked to their electronic band structures $E_{j}\left(\vec{k}\right).$ In semiconductors, this association allows for the information of electronic energy levels to be obtained by examining their optical properties [3,4,5]. While the absorption spectra $\alpha \left(E\right)$ are related to the transitions between valence and conduction bands, the shapes and intensities of $\alpha \left(E\right)$ are used for identifying CPs of materials where energy transitions occur with high probabilities. The imaginary part of $\tilde{n}\left(E\right)$ can be extracted via optical measurements by relating it to the density of states which reflect critical point features of the band structures. In the KK formalism, the real and imaginary parts of $\tilde{\varepsilon }\left(E\right)$ are expressed by the following relationships:

(A1a) $$\varepsilon_{1}\left(E\right)=1+\frac{2}{\pi }\int_{0}^{\infty }\frac{E\prime \varepsilon_{2}\left(E\prime \right)}{{\left(E\prime \right)}^{2}-{\left(E\right)}^{2}}\;dE\prime ,$$

(A1b) $$\varepsilon_{2}\left(E\right)=-\frac{2}{\pi }\int_{0}^{\infty }\frac{\varepsilon_{1}\left(E\prime \right)}{{\left(E\prime \right)}^{2}-{\left(E\right)}^{2}}\;dE\prime .$$

One must note that the optical properties of semiconductors play crucial roles in the design and functionality of different optoelectronic devices. Thus, from both technical and scientific standpoints it is important to derive simple analytical expressions of the optical constants ($\tilde{\varepsilon }\left(E\right),\tilde{n}\left(E\right)$). The method we have adopted here is based on an empirical scheme involving the inter-band transitions associated with the electronic band structures $E_{j}\left(\vec{k}\right).$ Following Adachi [5] we have approximated the dielectric function $\tilde{\varepsilon }\left(E\right)$ of binary hexagonal materials (InN, GaN) as a sum of terms involving the CPs. These terms provide major contributions to the model dielectric functions covering the optical response over the entire range of photon energies, E. The method can easily extend to ternary ${In}_{x}{Ga}_{1-x}N$ alloys. Next, we succinctly described the modified MDFs by including important energy gaps to illustrate the main dispersion mechanisms.

**The Modified Model Dielectric Function**

This approach offers a reasonably accurate description of the anisotropic behavior for optical constants of the binary hexagonal InN and GaN materials in the E⏊c polarization. The complex dielectric function $\;\tilde{\varepsilon }\left(E\right)$ has been expressed as the sum of terms corresponding to one-electron contributions from the main CP energies ($e.g.,\;E_{0}$, $E_{0\alpha }$, $E_{1\alpha }$ where α(≡A,B,C), and $E_{abi}$):

(A2) $$\tilde{\varepsilon }\left(E\right)={\tilde{\varepsilon }}_{0}\left(E\right)+{\tilde{\varepsilon }}_{0x}\left(E\right)+{\tilde{\varepsilon }}_{1}\left(E\right)+{\tilde{\varepsilon }}_{1x}\left(E\right)+\varepsilon_{\infty }.$$

In (A2), each energy-dependent term requires three fitting parameters: the energy, strength and broadening elements.

**Contribution of** $E_{0}$ **Gap**

The $E_{0}$ gap in the wz type semiconductors may be of the three-dimensional 3D $M_{0}$-type critical point in the BZ [5]. Assuming that the valence and conduction bands are parabolic, one can use the KK transformation to obtain the contribution of $E_{0}$ to $\tilde{\varepsilon }\left(E\right)\;$ as ${\tilde{\varepsilon }}_{0}\left(E\right)$ defined by:

(A3) $${\tilde{\varepsilon }}_{0}\left(E\right)=AE_{0}^{-\frac{3}{2}}f\left(\chi_{0}\right),$$

where

(A3a) $$f\left(\chi_{0}\right)=\chi_{0}^{-2}\left[2-{\left(1+\chi_{0}\right)}^{\frac{1}{2}}-{\left(1-\chi_{0}\right)}^{\frac{1}{2}}\right],$$

(A3b) $$\chi_{0}=\frac{E+i\Gamma_{0}}{E_{0}}\;,$$

The terms A, $E_{0}\;$ and $\Gamma_{0}\;i$n Equations (A3) and (A3b) signify, respectively, the oscillator strength, energy and damping constant of the main $E_{0}\;$ energy transition. Normally, it is required to calculate separate contributions from the $E_{0\alpha }$ (α≡A,B,C) CPs. Due to very low splitting energies among these energies, here $E_{0\alpha }$ is treated as singly degenerate (i.e., $E_{0}\approx E_{0\alpha }).$ Thus the excitonic contributions about the lowest energy at $E_{0}\approx E_{0\alpha }$ CPs are evaluated using [5]:

(A4) $${\tilde{\varepsilon }}_{0x\alpha }\left(E\right)=\sum_{\alpha =A,B,C}\left(\sum_{m=1}^{\infty }\frac{A_{0\alpha }^{ex}}{m^{3}}\frac{1}{E_{0\alpha }-\left(\frac{G_{0\alpha }^{3D}}{m^{2}}\right)-E-i\Gamma_{0}}\right),$$

where $A_{0\alpha }^{ex}$ is the three-dimensional (3D) exciton strength parameter $and\;G_{0\alpha }^{3D}$ is the 3D exciton binding energy.

**Contribution of** $E_{1\alpha }(\alpha \equiv A,B,C)$ **Gaps**

The structures found in the hexagonal InN in a region with energy higher than $E_{0}$ can be labeled as $E_{1\alpha }$ (α≡A,B,C) gaps. The $E_{1\alpha }$ CP energies may be of the 3D $M_{1}$-type. The $M_{1}$ CP longitudinal effective mass is usually much larger than its transverse counterparts, thus one can treat these CPs as two-dimensional 2D minima ${(M}_{0})$ [5]. The contribution of this type of 2D minima to $\tilde{\varepsilon }\left(E\right)$ can be expressed as:

(A5) $${\tilde{\varepsilon }}_{1}\left(E\right)=\sum_{\alpha =A,B,C}-B_{1\alpha }\chi_{1\alpha }^{-2}\ln\left(1-\chi_{1\alpha }^{2}\right),$$

(A6) $$\chi_{1\alpha }=\frac{E+i\Gamma_{1\alpha }}{E_{1\alpha }},$$

where $B_{1\alpha }$ and $\Gamma_{1\alpha }$ are the strengths and damping constants of the $E_{1\alpha }$ transitions, respectively. The Wannier-type 2D-$M_{0}$ exciton contributions to the critical point transitions $E_{1\alpha }$ can be written as:

(A7) $${\tilde{\varepsilon }}_{1x}\left(E\right)=\sum_{\alpha =A,B,C}\sum_{m=1}^{\infty }\frac{B_{1\alpha }^{ex}}{{\left(2m-1\right)}^{3}}\frac{1}{E_{1\alpha }-\left(\frac{G_{1\alpha }^{2D}}{{\left(2m-1\right)}^{2}}\right)-E-i\Gamma_{1\alpha }},$$

where $B_{1\alpha }^{ex}$ and $G_{1\alpha }^{2D}$ are the strengths and the binding energies of the excitons at $E_{1\alpha }$, respectively.

To explain the broadening caused by the electron–phonon and electron–impurities scattering, Kim et al. [88] have proposed replacing the damping constant Γ at critical point $E_{0}$ by the energy-dependent Gaussian broadening term Γ′(E) by assuming [88]:

(A8) $$\Gamma \prime \left(E\right)=\Gamma exp\left[-\alpha {\left(\frac{E-E_{c}}{\Gamma }\right)}^{2}\right]\;,$$

where α and Γ are adjustable model parameters. The term $E_{c}$ is the energy of the critical point at which transition occurs.

**Contribution of Higher**$E_{abi}$**energy Gaps**

(A9) $${\tilde{\varepsilon }}_{abi}\left(E\right)=\frac{S_{abi}}{E_{abi}^{2}-E^{2}-iE\Gamma_{abi}},$$

where $S_{abi}$, $E_{abi}$ and $\Gamma_{abi}$ are the strength, energy position and damping constant of the higher-energy oscillators, respectively. Thus, the dielectric function is adjusted to the following form:

(A10) $${\tilde{\varepsilon }}^{\prime }\left(E\right)=\tilde{\varepsilon }\left(E\right)+{\tilde{\varepsilon }}_{ab1}\left(E\right)+{\tilde{\varepsilon }}_{ab2}\left(E\right)+{\tilde{\varepsilon }}_{ab3}\left(E\right),$$

One must note that all the model parameters are carefully obtained using a least-square fitting algorithm, by minimizing the experimental and theoretical values of optical ${((\varepsilon }_{1}^{calc}(E),\varepsilon_{1}^{exp}(E);\varepsilon_{2}^{calc}(E),\varepsilon_{2}^{exp}(E))\;or$ ($n^{calc}(E),n^{exp}(E);\kappa^{calc}(E),\kappa^{exp}(E)$)) constants. The relative rms error obtained for the optical constants of GaN and InN is 5–7%. The modified MDF approach that we have described here in our simulations departs from those of Djurišić and Li [83] on two important fronts. First, our approach has used the correct value of $\;E_{0}$ ~0.7 eV for the wz InN [11,12,13] than the much higher value ($E_{0}$ ~2.247 eV) considered in [83]. Secondly, we have included higher-energy $E_{abi}$ contributions (Equation (A9)) around $\;E_{0}$ from the band structure calculations $E_{j}(\vec{k})$ (see Table 2). These changes enabled us to obtain a significant improvement in the optical constants.
